# Honey Geographical Origin Characterization and Authentication Based on Spectrophotometric Assays, Physicochemical Parameters, and LC-MS/MS Polyphenolic Profiling

**DOI:** 10.3390/foods14223828

**Published:** 2025-11-08

**Authors:** Danica Mostoles, Fleur de Krijger, Andrea Mara, Gavino Sanna, Javier Saurina, Sònia Sentellas, Oscar Núñez

**Affiliations:** 1 Department of Chemical Engineering and Analytical Chemistry, Universitat de Barcelona, Martí i Franquès 1-11, 08028 Barcelona, Spainxavi.saurina@ub.edu (J.S.); sonia.sentellas@ub.edu (S.S.); 2 Department of Chemical, Physical, Mathematical and Natural Sciences, University of Sassari, Via Vienna 2, 07100 Sassari, Italy; amara@uniss.it (A.M.); sanna@uniss.it (G.S.); 3 Research Institute in Food Nutrition and Food Safety, Universitat de Barcelona, Av. Prat de la Riba 171, Edifici Recerca (Gaudí), 08921 Santa Coloma de Gramenet, Spain; 4 Serra Húnter Fellow Programme, Generalitat de Catalunya, Via Laietana 2, 08003 Barcelona, Spain

**Keywords:** honey geographical origin, spectrophotometric antioxidant assays, physicochemical parameters, LC-MS/MS polyphenolic profiling, chemometrics

## Abstract

Honey is a widely consumed natural sweetener produced by honeybees from the nectar of plants, secretions of living parts of plants, or insect excretions. Its high value is due to its nutritional value and multiple benefits to human health. However, due to the diversity in geographical origins, the properties of honey can vary depending on the region of production, leading to discrepancies in honey pricing. Therefore, it is essential to examine these variations by analyzing several parameters in honey from diverse regions. In this work, honeys from eight countries were characterized by measuring several physicochemical parameters and spectrophotometric assays aiming at geographical origin authentication. In addition, the polyphenolic profile of the samples was obtained by LC-LRMS. An acceptable discrimination of the samples was obtained when considering all variables altogether, with classification errors lower than 31.9%.

## 1. Introduction

Honey is a widely consumed natural product widely appreciated by society as a sweetener for its organoleptic, nutritional, and health-beneficial properties [1,2]. The European Union Council Directive 2001/110/EC defines honey as “the natural sweet substance produced by *Apis mellifera* bees from the nectar of plants or from secretions of living parts of plants or excretions of plant-sucking insects on the living parts of plants, which the bees collect, transform by combining with specific substances of their own, deposit, dehydrate, store and leave in honeycombs to ripen and mature” [3]. A similar definition is provided by the Codex Alimentarius of the Food and Agriculture Organization (FAO) of the United Nations and the World Health Organization (WHO) [4]. Therefore, honey can be classified into two main groups according to the botanical source: blossom honey (produced from nectar) and honeydew honey (produced from plant secretions or plant-sucking insect excretions), with very different properties [5,6,7]. Honey is a quite complex mixture, comprising 70–80% of carbohydrates (with the monosaccharides glucose (around 31%) and fructose (around 38%) being the most abundant), 10–20% of water, and a great number of minor components coming from the plants, added by the honeybees, or produced in biochemical reactions during honey maturation in the hives [8,9]. The content and distribution of these minor bioactive substances, such as phenolic compounds, are strongly related to honey’s botanical and geographical origin; in addition, they are also mainly responsible for honey’s organoleptic attributes and some beneficial properties for human health, including antioxidant, anti-inflammatory, antifungal, and antibacterial activities [1,10].

Nowadays, there is a great diversity of honey products due to its worldwide production, which has led to the implementation of quality schemes such as Protected Designation of Origin (PDO) and Protected Geographical Indication (PGI) within the European Union framework, aimed at highlighting the unique characteristics of foodstuffs typically associated with specific regions of production [11]. In the case of honey, several PDOs and PGIs exist in each country. In Spain, for instance, there are currently seven PDOs and one PGI [12].

This scenario has led to a disparity in the consumer’s perception of the quality of honey, resulting in significant differences in product pricing. In addition, consumer perception varies greatly between multifloral and monofloral honeys, even considering some botanical varieties to be of higher quality than others. In addition, the societal demand for this product exceeds production, a situation that has recently been aggravated by the drought and environmental conditions in many honey-producing areas [13,14,15]. As a consequence, honey is today among the foodstuffs most vulnerable to manipulation for illicit economic benefits, finding mislabeling and adulteration among the most common honey frauds [16,17,18]. Given the significant variations in various properties of honey from different geographical or botanical origins, it is plausible to identify patterns that can differentiate honey samples into distinct groups. For instance, several studies have reported that honey from the same botanical origin but from different geographical origins exhibits significant differences in some physicochemical parameters (e.g., pH, conductivity, and moisture) [19,20,21,22,23]. Furthermore, differences in the honey phenolic composition could result in variations in antioxidant properties [23,24,25].

This work aims to characterize honey from different countries to find specific patterns that differentiate production regions. For such a purpose, 68 honey samples from eight countries (Spain, Italy, France, Portugal, Serbia, Japan, China, and Australia) were characterized by measuring several physicochemical parameters, including pH, conductivity, moisture, and Brix index. In addition, several spectrophotometric indexes were used for estimating total phenolic content, total flavonoid content, antioxidant capacity, and total reducing sugar content. The polyphenolic profile for each sample was also obtained by liquid chromatography coupled to low-resolution mass spectrometry (LC-LRMS). Each parameter was individually evaluated to identify significant differences and trends between the countries under study. Finally, sample classification was assessed using chemometrics, first exploiting separately the physicochemical parameters, the spectrophotometric assays, and LC-LRMS data, as well as dealing with data fusion considering all the data collected.

## 2. Materials and Methods

### 2.1. Chemicals and Reagents

Acetonitrile (UHPLC supergradient ACS), methanol (ChromosolvTM for HPLC, ≥99.9%), Folin–Ciocalteu reagent, and Al(NO_3_)_3_·9H_2_O were purchased from PanReac AppliChem (Barcelona, Spain); FeCl_3_, HCl (37%, *v*/*v*), sodium acetate, and NaOH were obtained from Merck (Darmstradt, Germany); Na_2_CO_3_ was from Probus S.A. (Badalona, Spain); Fe (III)-2,2,6-tripyridyl-s-triazine (TPTZ) was obtained from Alfa Aesar (Kandel, Germany); 3,5-dinitrosalicylic acid (97%) was from Thermo Fisher Scientific (Waltham, MA, USA); and formic acid (≥98%) was from Sigma-Aldrich (St Louis, MO, USA). Water was purified using an Elix 3 coupled to a Milli-Q system from Millipore Corporation (Bedford, MA, USA). The water was filtered using a nylon membrane (0.22 µm) incorporated into the Milli-Q system.

Up to 39 polyphenol standards were used for identification and quantification purposes (Table S1, Supplementary Materials).

### 2.2. Samples and Sample Treatment

In this study, a total of 68 honey samples from eight different countries were analyzed, encompassing several botanical species. The samples were randomly selected with respect to botanical origin, while ensuring that each country was adequately represented (see Table S2 in the Supplementary Materials). Melissopalynological analyses for Italian samples are also available in Table S3, in the Supplementary Materials.

For spectrophotometric assays and LC-MS/MS analyses, 1 g of honey was weighed into a 50 mL PTFE centrifuge tube and dissolved in 10 mL of 0.1% formic acid/methanol (80:20, *v*/*v*). The mixture was homogenized with a VibraMix Vortex (OVAN, Barcelona, Spain), and the resulting solution was sonicated for 10 min (5510 Branson ultrasonic bath, Barcelona, Spain). In addition, all the solutions were filtered with a 0.45 µm syringe membrane filter into 2 mL glass HPLC vials before injection into the LC-MS/MS system. For the measurement of pH and conductivity, 1.5 g of honey was weighed into a 50 mL PTFE centrifuge tube and dissolved in 15 mL of Milli-Q water by vigorously mixing in a VibraMix Vortex for 1 min, followed by sonication for 10 min. All the solutions were stored at 4 °C until the measurements.

For LC-MS/MS analyses, quality control (QC) solutions were prepared by mixing 50 µL of each honey solution.

### 2.3. Physicochemical Parameters

#### 2.3.1. pH and Conductivity

Honey pH was determined by using a Basic 20 pH meter from Crison Instruments (Barcelona, Spain), calibrated with buffer solutions at pH 4.0 and 7.0. The conductivity was determined using an EC-meter BASIC 30+ conductometer from Crison Instruments, previously calibrated with three standard solutions with conductivity values of 147 µS cm^−1^, 1413 µS cm^−1^, and 12.88 mS cm^−1^.

#### 2.3.2. Water and Brix Index

Honey Brix index and water content (both in *w*/*w* percentage) were simultaneously determined by using a portable RHB-90ATC refractometer from HHTEC Hanstronik (Heidelberg, Germany), calibrated with a Brix solution of 65%.

### 2.4. Spectrophotometric Methods

#### 2.4.1. Total Phenolic Content by Folin–Ciocalteu Index

The total phenolic content (TPC) in honey was estimated with the Folin–Ciocalteu (FC) assay as previously described [26]. Briefly, 250 µL of the honey solutions (or the gallic acid standard solutions) were mixed with 250 µL of the FC reagent in 8 mL amber vials. After 2 min, 2.5 mL of a 7.5% sodium carbonate solution was added and allowed to react for 20 min at 40 °C. Then, the reaction was stopped by placing the vials in an ice bath, and the absorbance was measured with an 8453 UV-Vis Spectrophotometer (Agilent, Santa Clara, CA, USA) at 756 nm with a disposable cuvette (10 mm optical path, from BRAND GMBH + CO KG, Wertheim, Germany). For calibration, gallic acid standard solutions were prepared with an acetonitrile/water (50:50 *v*/*v*) mixture within the concentration range 15 to 100 mg L^−1^, and the results were expressed as mg of gallic acid equivalents (GAE) per g of honey.

#### 2.4.2. Total Flavonoid Content by Aluminum Complexation Reaction

The total flavonoid content (TFC) in honey was determined by means of an aluminum complexation reaction as previously described [26], with some modifications. Briefly, 600 µL of honey solutions (or 100 µL of quercetin standard solution) were mixed with 100 µL of a 2% (*w*/*v*) Al(NO_3_)_3_ solution and 100 µL of a 1 M sodium acetate solution in an 8 mL amber vial. The mixture was then diluted to a total volume of 2.0 mL with the acetonitrile/water mixture (50:50 *v*/*v*), and the absorbance was measured with the spectrophotometer at 425 nm. For calibration, standard solutions of quercetin were prepared in the acetonitrile/water mixture (50/50 *v*/*v*) within the concentration range 100 to 1000 mg L^−1^. TFC results were expressed as mg of quercetin equivalents per g of honey.

#### 2.4.3. Antioxidant Activity by Ferric Reducing Antioxidant Power Assay

Honey antioxidant activity was estimated by means of the ferric reducing antioxidant power (FRAP) assay as previously described [26,27], with some modifications. The FRAP reagent was first prepared by mixing 20 mM FeCl_3_, 10 mM 2,4,6-tripyridyl-S-triazine (TPTZ, dissolved in 50 mM HCl solution), and 50 mM formic acid solution in a 1:2:10 (*v*/*v*/*v*) ratio. Then, 200 µL of honey solutions (or the Trolox standard solution) were mixed with 600 µL of the FRAP reagent in 8 mL amber vials and diluted to a final volume of 2.5 mL with Milli-Q water. After 5 min, the absorbance was measured at 595 nm. For calibration, standard solutions of Trolox were prepared in the acetonitrile/water mixture (50:50 *v*/*v*) within the concentration range 10 to 550 mg L^−1^. FRAP results were expressed as mg of Trolox equivalents per g of honey.

#### 2.4.4. Reducing Sugars by the 3,5-Dinitrosalycilic Acid (DNS) Assay

Total honey reducing sugars were estimated using the 3,5-dinitrosalicylic acid (DNS) spectrophotometric assay as previously described [28,29]. The DNS reagent was prepared by dissolving 5 g of 3,5-dinitrosalicylic acid in 400 mL of 0.5 M sodium hydroxide, followed by the dissolution of 150 g of sodium potassium tartrate and the addition of 100 mL of Milli-Q water. Then 1 mL of a 100-fold dilution of the honey solution (or glucose standard solution) was mixed with one droplet of HCl 37% in a 15 mL PTFE vial and maintained at 90 °C for 5 min to hydrolyze polysaccharides. Then, three droplets of a 5M potassium hydroxide solution and 3 mL of DNS reagent were added, and the reaction was allowed to develop at 90 °C for 5 min. The reaction was stopped by adding 6 mL of Milli-Q water, and the absorbance was measured at 540 nm. For calibration, standard solutions of glucose prepared in Milli-Q water within the concentration range 0.2 to 1.4 g L^−1^ were used. Results were expressed as mg of glucose equivalents per g of honey.

### 2.5. LC-MS/MS Polyphenolic Profiling

A total of 39 polyphenols were monitored by liquid chromatography coupled to tandem mass spectrometry in multiple reaction monitoring mode (MRM). The chromatographic separation was carried out on a Kinetex^®^ C18 (100 × 4.6 mm i.d., 2.6 µm partially porous particle size) fused-core column from Phenomenex (Torrance, CA, USA) in an Agilent 1100 Series HPLC system (Agilent Technologies, Palo Alto, CA, USA). The instrument was equipped with a binary pump (G1312A) and an automatic injector (G1367A) and coupled to an AB Sciex 4000 QTrap hybrid triple quadrupole/linear ion trap mass spectrometer (AB Sciex, Framingham, MA, USA). The flow rate for the chromatographic separation was 0.8 mL·min^−1^, and the mobile phase components consisted of a 0.1% formic acid aqueous solution (Solvent A) and acetonitrile (Solvent B). The elution program for the separation can be found in Table S4 (Supplementary Materials). The injection volume was 5 µL. Mass spectrometer conditions were optimized to work with electrospray ionization (ESI) in negative mode. Nitrogen was used as a curtain gas, ion source gas 1, and ion source gas 2, working at a flow of 10, 50, and 50 a.u. (arbitrary units), respectively. The ionization temperature and ionspray voltage were 400 °C and 2.5 kV, respectively. The list of the targeted polyphenols, together with the optimized MS/MS parameters, which include the declustering potential (DP), collision energy (CE), the collision cell exit potential (CXP), and the selected reaction monitoring (SRM) transitions, is summarized in Table S5, found in the Supplementary Materials.

All samples were analyzed randomly, injecting the QC solution every ten samples to study the reproducibility and robustness of the method.

### 2.6. Data Treatment

#### 2.6.1. Data Analysis

Data obtained from spectrophotometric assays and physicochemical parameters were subjected to statistical F and t tests to ascertain the significance of the observed differences. These statistical tests were carried out with Microsoft Excel (Microsoft, Redmond, WA, USA).

#### 2.6.2. Chemometric Analysis

Different combinations of data were contemplated, considering independently the spectrophotometric indexes, the physicochemical parameters, or the polyphenol signals, as well as all previous data combined.

For spectrophotometric assays and physicochemical properties, the corresponding data matrices of 68 samples × 4 variables (spectrophotometric or physicochemical parameters).

In the case of polyphenolics, the data matrix consisted of the peak areas for the detected polyphenols, with a dimension of 68 samples × 39 variables. Peak integration was performed with the Analyst software version 1.6.2 from AB Sciex (Framingham, MA, USA).

SOLO 8.6 software from Eigenvector Research (Manson, WA, USA) was used for data exploration by principal component analysis (PCA) and supervised classification by partial least squares-discriminant analysis (PLS-DA). The number of latent variables (LVs) for PLS-DA models was determined by the first minimum of the cross-validation (CV) error from the venetian blind. Classification parameters (e.g., sensitivity, specificity, and classification error) were studied to evaluate the classification performance of PLS-DA models. Sensitivity evaluates the capacity to correctly assign samples to their corresponding class and is calculated as TP/(TP + FN), with TP being the true positives and FN the false negatives; specificity evaluates the capacity of the model to correctly exclude samples not belonging to the group and is calculated as TN/(TN + FP), with TN being the true negatives and FP the false positives; the accuracy of the model is calculated as (TN + TP)/TS, with TS being the total number of samples.

## 3. Results and Discussion

### 3.1. Physicochemical Parameters

**Figure 1:**
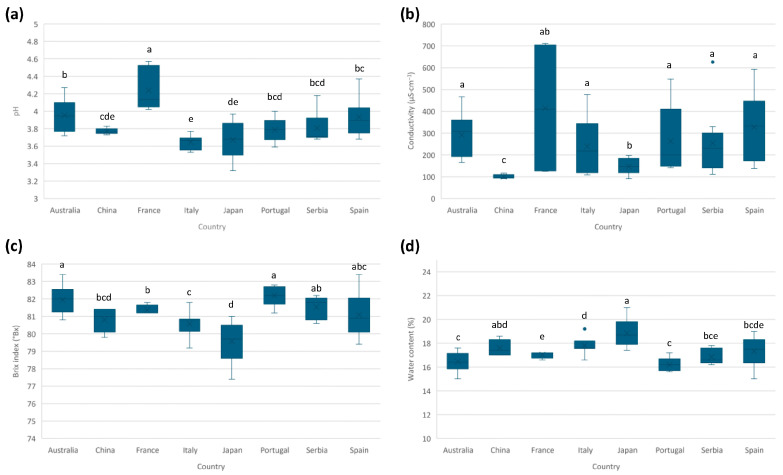
Boxplots for the studied physicochemical parameters and respective countries: (**a**) pH, (**b**) conductivity, (**c**) Brix index (°Bx), and (**d**) water content. The same letter indicates no significant differences (α = 0.05).

The composition of honey is highly complex, and its physicochemical properties (e.g., pH, conductivity, moisture, and Brix index) can vary depending on different attributes such as botanical variety and geographic setting. By analyzing and comparing the differences in the studied parameters, regional variations can be identified, providing insight into how the geographical origin of honey influences its composition. For this reason, in this section, differences in the physicochemical properties among the countries considered will be studied. In Table S6 (Supplementary Materials), individual and mean values for each studied physicochemical parameter are given. Additionally, Figure 1 shows the boxplots for each parameter and country.

The honey pH usually ranges from 3.5 to 6.5 [30], with this acidic pH mainly due to the presence of organic acids. The mean pH values for the analyzed honeys ranged from 3.6 to 4.2, in accordance with the Codex Alimentarius [4]. No significant differences were identified between the average values for most countries. However, some trends were observed (Figure 1a). Samples from France presented the highest pH values, with an average value significantly different from the rest of the countries. In contrast, Italian samples presented the lowest average value, but not significantly different from some other origins.

For conductivity, the average values ranged from 100 to 414 µS cm^−1^, in accordance with the Codex Alimentarius [4]. Samples from China presented the lowest conductivity (Figure 1b), with an average value statistically different from the rest of the countries. Therefore, this parameter may help to discriminate Chinese samples from the rest.

The Brix index provides an index of the honey sugar content, expressed as Brix degrees (°Bx), which is equivalent to g of sucrose per 100 g of honey. Brix index and water content were inversely correlated, observing that when values of sugar content were higher, the values of water content were lower. Usual values of sugar content in honey (expressed as the sum of fructose and glucose content) are higher than 60 °Bx and water content ≤20, according to the Codex Alimentarius [4]. Values summarized in Table S6 show that all honeys present sugar content between 79 and 82.2%; for the water content, these values are between 16.2 and 18.8%. Samples from Japan presented significantly lower sugar content and higher water content than those from the other countries.

### 3.2. Antioxidant Capacity and Reducing Sugars

In a previous work, several spectrophotometric assays of antioxidant capacity were applied to characterize and classify Spanish honeys [26]. These assays helped to understand the chemical composition of honey. The differences among sample groups were useful for honey characterization and authentication according to various features. In this work, antioxidant and radical scavenging tests were carried out, along with the spectrophotometric determination of reducing sugars to characterize and classify honeys from different countries. Table S7 in the Supplementary Materials gives the obtained results. Additionally, the correlation of the different antioxidant tests was also performed.

#### 3.2.1. Total Phenolic Content (TPC)

**Figure 2:**
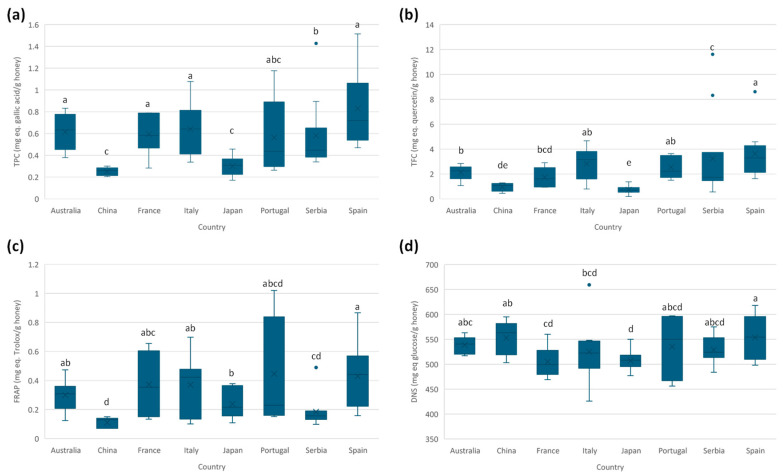
Boxplots for the measured spectrophotometric assays according to the country of production: (**a**) total phenolic content (TPC), (**b**) total flavonoid content (TFC), (**c**) ferric reducing agent power (FRAP), and (**d**) reducing sugars by 3,5-dinitrosalicylic acid assay (DNS). Countries with the same letter indicate no significant differences (α = 0.05).

The total phenolic content (TPC) expressed as mg of gallic acid equivalents per gram of honey is estimated by the Folin–Ciocalteu assay, as described in the Section 2.4.1. Comparing the results obtained (see Table S7 in the Supplementary Materials), mean values ranged between 0.25 and 0.8 mg GAE per gram of honey, similar to those reported previously in the literature on Spanish honeys [26] as well as honey from diverse botanical origins [31,32]. Figure 2a shows the boxplots for each country. A significant dispersion is observed in some sample groups (such as Spain, Italy, Portugal, France, and Australia), which can be explained by the diversity of botanical varieties considered in these sample groups (Table S2, Supplementary Materials). This fact results in greater variability compared to the other sample groups, which consider fewer botanical varieties.

#### 3.2.2. Total Flavonoid Content (TFC) 

The total flavonoid content (TFC) was estimated by an aluminum complexation reaction (see Section 2.4.2). TFC average values ranged between 0.7 and 3 mg quercetin equivalents per gram of honey, which are in accordance with previous results [26]. Figure 2b shows the TFC boxplots of each country of production. Samples from Japan present the lowest mean value, but it was not significantly different from Chinese honey.

#### 3.2.3. Ferric Reducing Antioxidant Power (FRAP)

The ferric reducing antioxidant power (FRAP) assay is used to estimate the antioxidant capacity of the samples. Average values for FRAP ranged between 0.11 and 0.5 mg Trolox equivalents per gram of honey, similar to those obtained in the characterization of Spanish honey [26] and honey from diverse botanical origins [33]. Figure 2c shows the boxplots. A greater dispersion is observed for samples from Spain, Italy, Portugal, and France, probably because of the wide range of botanical varieties. Due to this huge dispersion, no significant differences are observed among countries, although samples from China and Serbia tend to have lower FRAP values.

#### 3.2.4. Correlation Between Antioxidant Assays

The correlation between antioxidant assays was studied, as the information obtained from each assay may be complementary.

**Figure 3:**
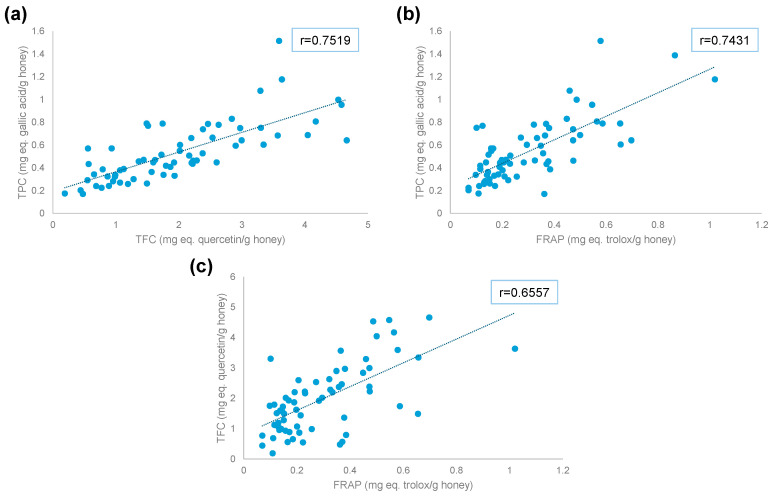
Correlation between antioxidant indexes: (**a**) TFC vs. TPC, (**b**) FRAP vs. TPC, and (**c**) FRAP vs. TFC.

When comparing TPC and TFC, similar trends are identified. Thus, samples from Japan and China present low TPC and TFC, while samples from Spain, Italy, and Portugal exhibit higher values. Figure 3a shows the correlation between TPC and TFC indexes, with a correlation coefficient of 0.7519 (*p* < 6 × 10^−13^).

Figure 3b shows the correlation between FRAP and TPC, with a correlation coefficient of 0.7431 (*p* < 9 × 10^−13^), indicating a positive correlation between the two variables. The correlation coefficient between FRAP and TFC (Figure 3c) was 0.6557 (*p* < 4 × 10^−9^). In this case, most samples present higher values of TFC but lower values of FRAP. This phenomenon is observed mostly in samples from Serbia. In addition, some samples from Portugal and France present higher FRAP but lower TFC.

Correlation values suggest complementary information between the assays studied, since the reactions performed in each assay were different.

#### 3.2.5. Reducing Sugars

The 3,5-dinitrosalicylic acid (DNS) assay is a spectrophotometric method for the quantification of reducing sugars and is frequently used in the food industry for quality control. This method provides a quick and easy determination of reducing sugars; however, other reducing species could interfere with the analysis, resulting in inaccurate results. As shown in Table S2 (Supplementary Materials), the average values ranged from 500 to 550 mg of glucose equivalents per gram of honey. These results are similar to the ones reported in the literature [34]. Figure 2d shows the boxplots of the DNS assay. Most of the honeys from the countries under study presented similar contents of reducing sugars.

### 3.3. Polyphenolic Profiling by LC-MS/MS

The polyphenolic profile to characterize the honeys was obtained using LC-MS/MS in multiple reaction monitoring (MRM) mode, using the obtained peak signals as chemical descriptors for each country. The analyses performed can identify chemical markers for each country, which may be helpful in authenticating geographical origin. Table 1 summarizes the polyphenols detected in each country.

Up to 39 polyphenols were monitored in this study, of which 23 were detected in the honeys. Among them, 3,4- and 4-hydroxybenzoic acids, *trans*-cinnamic acid, and pinobanksin are common in all the samples. In addition, there are some compounds that were detected in a few countries. For instance, caffeic acid is present in honey from France, Serbia, and Spain; hesperidin in samples from China, Japan, Portugal, and Spain; naringenin in samples from France, Serbia, and Spain; naringin in samples from Japan and Portugal; and 3-hydroxytyrosol in samples from Portugal and Spain. Some compounds were detected in only one country, which could serve as specific chemical markers to discriminate honeys from that country (although their presence was not general and they were only found in some samples). For example, quercetin was detected in some samples from Australia, rutin was detected in some samples from Portugal, and chrysin was detected in some samples from Serbia, as previously referred to in the literature [35].

Furthermore, although it is not the main aim of the present study, certain correlations between the identified polyphenols and the botanical varieties of the analyzed honeys can be established. For instance, naringin was detected, even though in a minority of samples, in those countries where blossom honeys are present (see Table S2 in the Supplementary Materials), which is in accordance with results reported in the literature [36]. Similarly, ferulic acid, previously identified in linden honey samples [37], was specifically detected in a limited number of samples from Serbia, which was the only country that considered honeys from this botanical variety. In addition, some correlations between the climatic conditions and the identified polyphenols can also be recognized. For example, 3-hydroxytyrosol and hesperidin were highly detected in Portuguese and Spanish samples, which share similar climatic conditions. Another example can be observed in samples from Serbia and France, which share certain identified polyphenols, such as pinocembrin or epigallocatechin, as well as temperate climatic conditions, and are based on the Köppen–Geiger climatic classifications [38]. Together, these findings suggest that these compounds may be characteristic of honeys produced in countries with such climate conditions. In any case, it would be necessary to carry out a more detailed study by increasing the number of samples of the same botanical variety but produced in different countries, or in specific climatic zones, to strengthen the possible correlations with the detected polyphenols.

### 3.4. Chemometric Data Analysis for Geographical Origin Discrimination

Even though some tendencies can be identified when comparing the respective mean values of parameters determined above, perfect discrimination was not accomplished. For this reason, chemometric analysis was considered to authenticate the geographical origin of the studied honeys. The following cases were examined: (i) physicochemical parameters, (ii) spectrophotometric assays, (iii) LC-MS/MS polyphenolic profiling, and (iv) all parameters, performing a data fusion.

#### 3.4.1. Classification of Honey Origin Using Physicochemical Parameters

It was observed that, for all physicochemical parameters, even though some tendencies were found in the boxplots and some class differences were statistically significant, a full geographical discrimination was not achieved based on these attributes alone. Thus, multivariate data analysis was performed to assess whether the joint study of these variables could accomplish country discrimination of honey.

**Figure 4:**
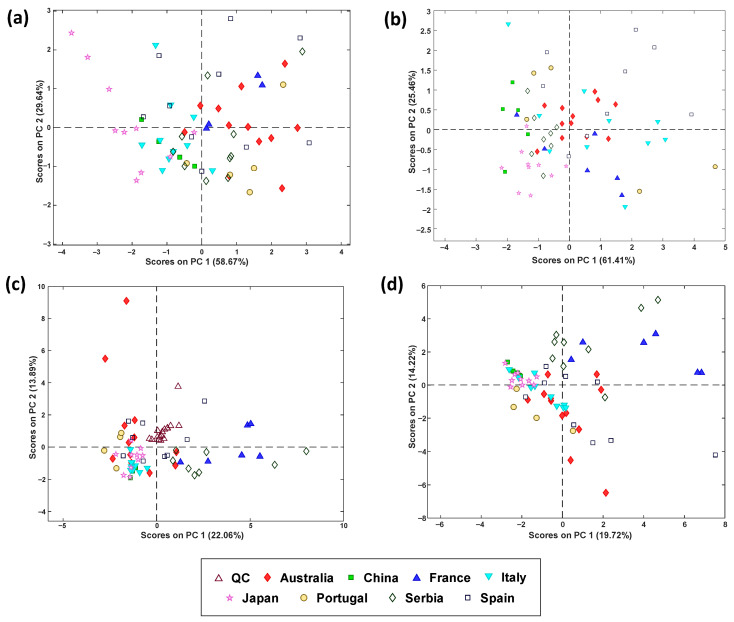
Principal component analysis (PCA) scores plots (PC1 vs. PC2) for the different variable groups: (**a**) physicochemical parameters, (**b**) spectrophotometric assays, (**c**) LC-MS/MS polyphenolic profiling, and (**d**) data fusion.

A PCA model was built to study the natural tendencies and grouping of the samples. Figure 4a depicts the scores plot on PC2 vs. PC1, where some trends in the different sample groups can be observed, even though overlap of some classes persisted. For instance, samples from Japan and Italy present negative values of PC1, while samples from Portugal present negative values of PC2. To study the sample classification performance, a PLS-DA model was built, needing only one latent variable (LV). In Table 2, observed values of sensitivity (between 40 and 100%) tend to be higher than the values of specificity (between 40 and 83.9%), meaning that most samples are correctly classified to their corresponding country, while other samples are incorrectly assigned to other countries, subsequently resulting in classification errors between 14.8 and 50.5%. This moderate performance suggests that the descriptive capacity of the measured parameters is limited and, therefore, some samples are misclassified. In the case of Spain, lower values of sensitivity were obtained, meaning that the measured parameters are more similar to the samples of other countries, suggesting again that the measured physicochemical parameters were not adequate to discriminate samples from this country. Japanese samples were better discriminated from the rest of the countries, since cross-validated values of sensitivity, specificity, and classification error were 80%, 83.9%, and 18%, respectively. This finding suggests that samples from this country present unique values of the measured physicochemical parameters.

In summary, the classification based on simple parameters such as the ones considered in this section seems to be useful to discriminate some countries from the rest; however, additional information may be needed to improve the classification rates.

#### 3.4.2. Classification of Honey Origin Using Spectrophotometric Indexes

Some differences in the spectrophotometric data were observed among the countries considered in this contribution. However, clear country discrimination was not observed when considering these attributes alone. For this reason, multivariate data analysis was performed with the aim of studying the classification performance when considering simple spectrophotometric assays, as those considered in this section.

A remarkable class overlapping was noted when observing the PCA scores plot (PC1 vs. PC2) (Figure 4b). For instance, samples from China, Serbia, and Japan display negative values of PC1, samples from Spain are represented by positive PC2 values, and French samples are represented by negative PC2 values; however, clear tendencies are not identified for the rest of the countries. To study the classification performance of the samples, a PLS-DA model was built with two latent variables, and the classification outcomes are given in Table 3. Cross-validated values of sensitivity, specificity, and classification error range between 50 and 100% (except for Portuguese samples), 49.1 and 85.5%, and 10.8 and 59.2%, respectively. Japan and China classes present the highest sensitivity and specificity, meaning that most samples are correctly assigned and have unique properties, excluding samples not belonging to these countries. On the contrary, for Australia, France, and Portugal, a worse classification performance is obtained, with higher values of specificity than sensitivity (i.e., although samples from these countries are incorrectly assigned to other countries, samples from other countries are successfully excluded from these countries). Overall, these simple spectrophotometric data may not provide sufficient discriminatory features for effective classification of countries, given the low sensitivity values obtained for Australian and Portuguese samples.

#### 3.4.3. Classification of Honeys Based on LC-MS/MS Polyphenolic Profiling

The potential of the identified phenolic compounds as chemical markers to address honey geographical origin authentication was studied. For that purpose, PCA and PLS-DA models were constructed using the polyphenolic profiles.

Figure 4c shows the PCA scores plot on PC 1 vs. PC 2, and Table 4 summarizes the classification results from PLS-DA. The scores plot demonstrated that the method was reproducible and robust since quality controls (QCs) are clustered in the same region. In addition, some trends can be identified in samples from most countries. For instance, French and Serbian honeys present positive values of scores on PC1, while Japanese, Portuguese, Italian, and Chinese samples present negative values on this PC. For the Spanish and Australian samples, clear trends could not be identified. Regarding the classification, sensitivity, specificity, and classification errors between 50 and 100%, 61.1 and 100%, and 1.7 and 34.4%, respectively, were obtained. A poorer classification performance was observed for Australian honey, while Portuguese ones present better discrimination.

**Figure 5:**
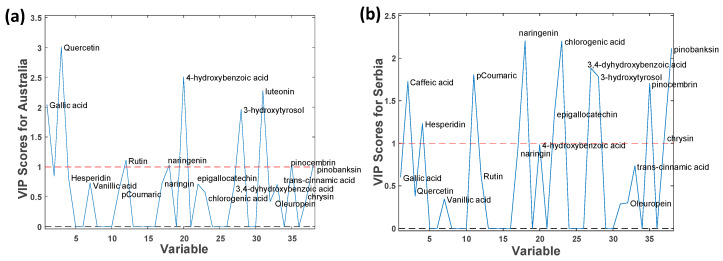
Partial least squares—discriminant analysis (PLS-DA) variable importance projection (VIP) plots when using LC-MS/MS polyphenolic profiles as chemical markers for (**a**) Australia and (**b**) Serbia.

The study of relevant variables for each country is useful to understand which phenolic compounds are more suitable for their geographical discrimination. As an example, the variable importance projection (VIP) scores plot for Australia and Serbia can be found in Figure 5, while the VIP Scores for the rest of the countries are in Figure S1 in the Supplementary Materials. As commented previously, quercetin was mostly detected in Australian samples, and chrysin was specific to Serbia. Hence, quercetin presents a high relevance for Australian VIPs, while this compound was not detected in other countries (see Table 1). However, since this compound was not detected in all Australian samples, its absence does not necessarily indicate another country. Another interesting compound is 3-hydroxytyrosol, relevant for Chinese, Italian, Japanese, Portuguese, and Spanish samples, and irrelevant for the rest of the countries.

#### 3.4.4. Data Fusion for Honey Classification

Considering that a good classification performance is not accomplished for all the countries using the individual set of parameters, data fusion with the studied parameters has been performed, aiming to improve the classification performance.

When observing the natural tendencies of the samples on the scores on PC1 vs. scores on PC2 plot obtained from the PCA model (Figure 4d), it is observed that samples from China, Japan, and Italy present negative values of scores on PC1 and samples from France and Serbia present positive values of scores on PC2, while Portuguese and most of Spanish and Australian samples are plotted on negative values. When studying the classification performance (Table 5) obtained from the PLS-DA model, which needed two latent variables, it was observed that cross-validated sensitivity values ranged from 75.0% to 100% (except for Spanish samples), specificity values ranged from 56.4% to 89.8%, and classification error values ranged from 10.9% to 41.2%. In general, acceptable classification values were obtained compared to the ones obtained in previous models, being able to successfully classify most of the samples into their corresponding class. However, for samples from Spain, values of sensitivity are lower compared to the other countries, meaning that some samples from this country have been incorrectly classified as other countries, probably due to similarities in most of the studied parameters with those obtained from samples from other countries.

Overall, it was observed that, when performing data fusion with the studied parameters, the classification performance has improved for some countries, such as Japan and Italy. However, when observing the rest of the countries, it can be concluded that the classification performance is better when considering a reduced number of variables, with most of them presenting a better classification performance when using LC-MS/MS polyphenolic profiles as chemical markers, suggesting that LC-MS/MS polyphenolic profiling alone may be sufficient for accurate classification. For the case of Spain, lower classification error values were obtained when using data from spectrophotometric assays, meaning that these assays were useful for the classification of samples from this country. However, when considering the overall performance, it can be concluded that, when aiming to characterize and classify honey from diverse geographical origins, LC-MS/MS polyphenolic profiling seems to be more adequate, since better classification performance is obtained. In addition, it is important to consider that, even though the rest of the measured properties are simpler, the proposed LC-MS/MS method offers a more time-efficient approach for characterizing a large set of samples.

## 4. Conclusions

Thorough characterization was performed on honey from eight countries, measuring several properties, including physicochemical parameters, spectrophotometric assays (measuring antioxidant properties and reducing sugars), and LC-MS/MS polyphenolic profiling, with the aim of understanding the influence of geographical origin on the measured properties of honey.

Although the individual analysis of physicochemical parameters and spectrophotometric assays did not reveal significant differences for most countries, their combination in multivariate data analysis provided acceptable classification performance. Classification errors were relatively high when using only physicochemical parameters or spectrophotometric antioxidant indexes (up to 60% and 50%, respectively). This finding suggests that these features may be strongly influenced by botanical origin or other intrinsic honey attributes than by geographical origin alone.

In addition, the phenolic compounds monitored through LC-MS/MS were largely similar across most countries. However, some specific compounds were identified on samples from certain origins, such as quercetin for Australian samples and chrysin for some Serbian samples. Most common polyphenols identified in samples from all countries included pinobanksin, *trans*-cinnamic acid, and 3,4- and 4-hydroxybenzoic acids. When using the monitored polyphenols as chemical descriptors, acceptable classification performance was achieved, with error values between 2.7 and 28.8% (except for Japan).

When performing a data fusion with all the measured properties, classification error values between 10.8 and 26.7% (except for Spanish samples) were obtained, observing a significant improvement in the classification of the samples compared to the previously built models.

Overall, the findings in this study were useful to understand the chemical composition of honey and how it differs based on its geographical origin, which is useful to identify key parameters to assess honey authenticity using simple parameters.

## Figures and Tables

**Table 1:** Polyphenolic compounds detected in the honey samples: AU: Australia; CH: China; FR: France; IT: Italy; JP: Japan; PT: Portugal; SE: Serbia; SP: Spain. Detected compounds in >60% samples are marked in green (+), those detected in <30% of the samples in orange (-/+), those present in the range of 30–60% of the samples in yellow (+/-), and those not detected in red (-).

Family	Compound	AU	CH	FR	IT	JP	PT	SE	SP
Phenolic acids	2,5-dihydroxybenzoic acid	-	-	-	-	-	-/+	-	-
	3,4-dihydroxybenzoic	+	+	+	+	+	+	+	+
	4-hydroxybenzoic acid	+	+	+	+	+	+	+	+
	Caffeic acid	-	-	+	-	-	-	+	+
	Caftaric acid	-	-	-	-	-	-	-	-
	Chlorogenic acid	+/-	-/+	+/-	-	+/-	-/+	+	+/-
	Ferulic acid	-	-	-	-	-	-	-/+	-
	Gallic acid	+	-/+	+	+/-	+/-	+	-/+	+/-
	*p*-Coumaric acid	-/+	-	+	-	-/+	-/+	+/-	+/-
	Syringic acid	-	-	-	-	-	-	-	-
	Vanillic acid	-/+	-	-	-	-	-	-	-/+
	Trans-cinnamic acid	+	+	+	+	+	+	+	+
Flavonoids	Apigenin	-	-	-	-	-	-	-	-/+
	Catechin	-	-	-	-	-	-	-	-
	Chrysin	-	-	-	-	-	-/+	+/-	-/+
	Epicatechin	-	-	-	-	-	-	-	-
	Galangin	-	-	-	-	-	-	-	-
	Hesperetin	-	-	-	-	-	-	-	-
	Hesperidin	-/+	+/-	-	-/+	+/-	+	-	+
	Kaempferol	-	-	-	-	-	-	-/+	-
	Luteonin	-/+	-	-	-	-	-	-	-
	Myricetin	-	-	-	-	-	-	-	-
	Naringenin	-/+	-	+	-/+	-	-/+	+	+/-
	Naringin	-/+	-	-	-	+/-	+	-	-/+
	Pinobanksin	+	+	+	+/-	+	+	+	+
	Pinocembrin	-/+	-	+	-	-	-/+	+	-/+
	Quercetin	+/-	-	-	-	-	-	-	-
	Rutin	-/+	-	-	-	-/+	+/-	-	-
Stilbenes	Resveratrol	-	-	-	-	-	-	-	-
Other polyphenols	3-hydroxytyrosol	-/+	-	-	-/+	-/+	+	-	+
	3-methylcatechol	-	-	-	-	-	-	-	-
	4-methylcatechol	-	-	-	-	-	-	-	-
	Catechol	-	-	-	-	-	-	-	-
	Epigallocatechin	-	-	+	-	-	-	+/-	-
	Ethyl gallate	-	-	-	-	-	-	-	-
	Oleuropein	-	-	-	-	-/+	-	-	-
	Vanillin	-	-	-	-	-	-	-	-

**Table 2:** Values of sensitivity, specificity, and classification error for the multiclass PLS-DA model when using physicochemical parameters as chemical descriptors for the classification. Cal: Calibration; CV: Cross-validation.

Class	Sensitivity (%)		Specificity (%)		Class. Error (%)	
	Cal	CV	Cal	CV	Cal	CV
**Australia**	83.3	83.3	68.5	68.5	24.1	24.1
**China**	100.0	100.0	49.2	49.2	25.4	25.4
**France**	100.0	75.0	56.5	59.7	21.8	32.7
**Italy**	90.0	90.0	55.4	57.1	27.3	26.4
**Japan**	90.0	80.0	80.4	83.9	14.8	18.0
**Portugal**	80.0	80.0	57.4	59.0	31.3	30.5
**Serbia**	80.0	70.0	39.3	44.6	40.4	42.7
**Spain**	50.0	40.0	62.5	58.9	43.8	50.5

**Table 3:** Values of sensitivity, specificity, and classification error for the multiclass PLS-DA model when using spectrophotometric assay data for the classification. Cal: Calibration; CV: Cross-validation.

Class	Sensitivity (%)		Specificity (%)		Class. Error (%)	
	Cal	CV	Cal	CV	Cal	CV
**Australia**	0.0	58.3	86.8	62.3	56.6	39.7
**China**	100.0	100.0	81.7	78.3	9.2	10.8
**France**	50.0	50.0	66.1	66.1	41.9	41.9
**Italy**	70.0	60.0	70.9	67.3	29.5	36.4
**Japan**	90.0	90.0	89.1	85.5	10.5	12.3
**Portugal**	40.0	0.0	85.0	81.7	37.5	59.2
**Serbia**	100	100	49.1	49.1	25.4	25.4
**Spain**	66.7	66.7	78.6	78.6	27.4	27.4

**Table 4:** Values of sensitivity, specificity, and classification error for the multiclass PLS-DA model when using targeted polyphenols through LC-MS/MS as descriptors for the classification. Cal: Calibration; CV: Cross-validation.

Class	Sensitivity (%)		Specificity (%)		Class. Error (%)	
	Cal	CV	Cal	CV	Cal	CV
**Australia**	50.0	50.0	100.0	92.3	25.0	28.8
**China**	100.0	100	74.6	79.7	12.7	10.2
**France**	83.3	100	91.4	89.7	12.6	5.2
**Italy**	90.0	90.0	63.0	66.7	23.5	21.7
**Japan**	100.0	70.0	64.8	61.1	17.6	34.4
**Portugal**	100.0	100.0	96.7	95.0	1.7	2.5
**Serbia**	100.0	100.0	96.4	94.6	1.8	2.7
**Spain**	77.8	66.7	87.3	87.3	17.5	23.0

**Table 5:** Values of sensitivity, specificity, and classification error for the multiclass PLS-DA model when performing data fusion. Cal: Calibration; CV: Cross-validation.

Class	Sensitivity (%)		Specificity (%)		Class. Error (%)	
	Cal	CV	Cal	CV	Cal	CV
**Australia**	75.0	75.0	69.8	71.7	27.6	26.7
**China**	100.0	100.0	73.3	71.7	13.3	14.2
**France**	83.3	83.3	91.5	89.8	12.6	13.4
**Italy**	100.0	100.0	60.0	56.4	20	21.8
**Japan**	100.0	70.0	85.5	89.1	7.3	20.4
**Portugal**	100.0	75.0	86.9	88.5	6.6	18.2
**Serbia**	100.0	88.9	89.3	89.3	5.4	10.9
**Spain**	55.6	44.4	76.8	73.2	33.8	41.2

## Data Availability

Data are available upon request to the authors.

## Supplementary Materials

The following supporting information can be downloaded at: https://www.mdpi.com/article/10.3390/foods14223828/s1, Figure S1: Partial Least Squares Discriminant Analysis (PLS-DA) variable importance projection (VIP) plot for countries under study when using LC-MS/MS polyphenolic profiling as chemical markers: (a) China, (b) France, (c) Italy, (d) Japan, (e) Portugal, (f) Spain; Table S1: Standards of the analyzed phenolic compounds and their corresponding suppliers; Table S2: Honey geographical and botanical origin information; Table S3: Distribution of Italian honey samples and melissopalynological analysis information; Table S4: Tandem mass spectrometry parameters for each polyphenol monitored in this study; Table S5: Gradient elution conditions for the LC-MS/MS chromatographic separation; Table S6: Physicochemical parameters for the analyzed honeys from each country; Table S7: TPC, TFC, antioxidant capacity (FRAP), and sugar content of the honeys under study.
