# Supplementary material for: Honey Geographical Origin Characterization and Authentication Based on Spectrophotometric Assays, Physicochemical Parameters, and LC-MS/MS Polyphenolic Profiling
Source: Foods. 2025 Nov 8;14(22):3828. doi: 10.3390/foods14223828 (PMC12651041; doi:10.3390/foods14223828)
Supplement: Supplementary file 1 [file foods-14-03828-s001.zip › foods-3944087-supplementary.pdf]

Supplementary Material

Honey Geographical Origin Characterization and Authentication Based on Spectrophotometric Assays, Physicochemical Parameters, and LC-MS/MS Polyphenolic Profiling

Danica Mostoles [1,*], Fleur de Krijger [1], Andrea Mara [2], Gavino Sanna [2], Javier Saurina [1,3], Sònia Sentellas [1,3,4] and Oscar Núñez [1,3,4,*]

[1] Department of Chemical Engineering and Analytical Chemistry, Universitat de Barcelona, Martí i Franquès 1-11, 08028 Barcelona, Spain; xavi.saurina@ub.edu (J.S.); sonia.sentellas@ub.edu (S.S.)
[2] Department of Chemical, Physical, Mathematical and Natural Sciences, University of Sassari, Via Vienna 2, 07100 Sassari, Italy; amara@uniss.it (A.M.); sanna@uniss.it (G.S.)
[3] Research Institute in Food Nutrition and Food Safety, Universitat de Barcelona, Av. Prat de la Riba 171, Edifici Recerca (Gaudí), 08921 Santa Coloma de Gramenet, Spain
[4] Serra Húnter Fellow Programme, Generalitat de Catalunya, Via Laietana 2, 08003 Barcelona, Spain
* Correspondence: dorcinmo7@alumnes.ub.edu (D.M.); oscar.nunez@ub.edu (O.N.)

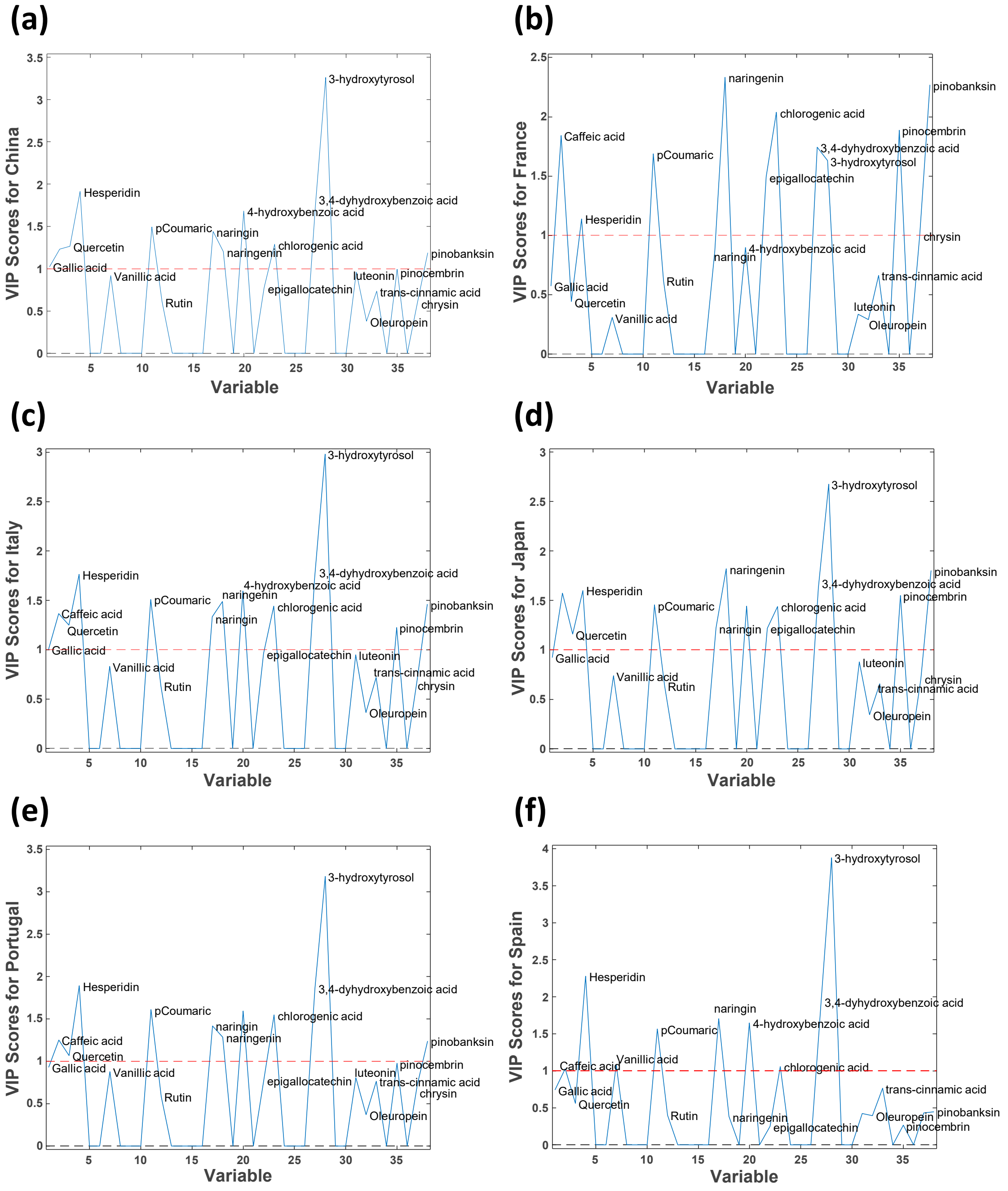

Figure S1. Partial Least Squares Discriminant Analysis (PLS-DA) variable importance projection (VIP) plot for countries under study when using LC-MS/MS polyphenolic profiling as chemical markers: (a) China, (b) France, (c) Italy, (d) Japan, (e) Portugal, (f) Spain.

Table S1. Standards of the analyzed phenolic compounds and their corresponding suppliers.

**Standards**	**Supplier**
2,5-dihydroxybenzoic, 3,4-dihydroxybenzoic, 4-hydroxybenzoic, caffeic, caftaric, ferulic, gallic, *p*-coumaric, syringic, and vanillic acids, apigenin, (-)-epicatechin, galangin, luteolin, polydatin, resveratrol, 3-methylcatechol, 4-methylcatechol, catechol, ethyl gallate, and oleuropein	Sigma-Aldrich (St. Louis, MO, USA)
(+)-Catechin, 3-hydroxytyrosol, myricetin, and rutin	TCI (Tokyo, Japan)
Chlorogenic acid, chrysin, pinobanksin and quercetin	Merck (Darmstadt, Germany)
(-)-Epigallocatechin and naringenin	Biosynth Carbosynth (Berkshire, United Kingdom)
Pinocembrin	Thermo Fisher Scientific (Waltham, MA, USA)
Hesperidin and hesperetin	Glentham (Wiltshire, United Kingdom)
Naringin	TargetMol (Boston, MA, USA)
Kaempferol, *trans*-cinnamic acid, vanillin	Fluka (Madrid, Spain)

Table S2. Honey geographical and botanical origin information.

Continent	Country	Total samples	Botanical variety	Number of samples
Asia	China (CH)	5	Acacia	1
			Milk Vetch	2
			Not provided	2
	Japan (JP)	10	Acacia	2
			Multifloral	2
			Wild rose	1
			Not provided	5
Europe	France (FR)	6	Acacia	2
			Chestnut	2
			Multifloral	2
	Italy (IT)	10	Eucalyptus	2
			Rosemary	2
			Multifloral	6
	Portugal (PT)	5	Heather and Chestnut	1
			Orange blossom	2
			Wild lavender	2
	Serbia (SE)	10	Acacia	2
			Forest	1
			Linden	2
			Meadow	3
			Multifloral	1
			Not provided	1
	Spain (SP)	10	Almond	2
			Blossom	2
			Eucalyptus	2
			Multifloral	2
			Rosemary	2
Oceania	Australia (AU)	12	Apple box	1
			Clover	1
			Fennel	1
			Ironbark	1
			Leatherwood	1
			Leptospermum	2
			Macadamia	1
			Manuka	1
			Multifloral	1
			Murdunna	1
			Orange blossom	1

Table S3. Distribution of Italian honey samples and melissopalynological analysis information

Country of origin	Number of samples	Botanical species	Number of samples	Pollen species	Threshold for the classification (pollen %)
Italy	10	Multifloral	6	*Vary according to seasonality and provenance, some common pollens are Asfodelus spp., Echium spp., Trifolium spp., Rosmarinus Galactites tormentosa, and Cistus spp*	No prevalent pollen species
		Rosemary	2	*Salvia rosmarinus*	10-50%
		Eucalyptus	2	*Eucalyptus camaldulensis, E. globulus, and E. viminalis*	> 90%

Table S4. Gradient elution conditions for the LC-MS/MS chromatographic separation

Time (min)	% of Acetonitrile	Elution
0-1	5-10	Linear gradient
1-4	10-16	Linear gradient
4-8	16	Isocratic
8-8.5	16-25	Linear gradient
8.5-13.5	25-60	Linear gradient
13.5-16	60-100	Linear gradient
16-16.5	100	Isocratic
16.5-16.6	100-5	Linear gradient
16.6-22	5	Isocratic; column equilibration

Table S5. Tandem mass spectrometry parameters for each polyphenol monitored in this study

Compound	Precursor ion (m/z)	Product ion (m/z)	Declustering potential (DP, V)	Collision energy (CE, V)	Cell exit potential (CXP, V)
**Gallic acid**	169	125	-40	-22	-19
**Caffeic acid**	179	135	-45	-20	-1
**Quercetin**	301	151	-80	-32	-1
**Hesperidin**	609	301	-115	-36	-19
**Resveratrol**	227	143	-70	-34	-1
**Ferulic acid**	193	134	-30	-22	-1
**Vanillic acid**	167	152	-65	-20	-1
**Ethyl gallate**	197	124	-60	-34	-3
**(+)-Catechin**	289	109	-110	-42	-19
**(-)-Epicatechin**	289	125	-85	-33	-7
**p-Coumaric acid**	163	119	-60	-18	-1
**Rutin**	609	300	-95	-48	-23
**Myricetin**	317	151	-95	-40	-9
**Syringic acid**	197	121	-40	-26	-9
**Astilbin**	449	285	-100	-32	-1
**Caftaric acid**	311	179	-50	-22	-11
**Diosmin**	607	299	-105	-34	-23
**Hesperetin**	301	286	-105	-36	-5
**Naringin**	579	271	-140	-36	-5
**Naringenin**	271	151	-90	-32	-25
**Catechol**	109	91	-25	-26	-5
**4-Hydroxybenzoic acid**	137	93	-55	-18	-7
**Ellagic acid**	301	284	-95	-98	-1
**Vanillin**	151	136	-50	-14	-31
**(-)-Epigallocatechin**	305	125	-80	-26	-25
**Chlorogenic acid**	353	191	-60	-20	-17
**3-Methylcatechol**	123	108	-50	-22	-13
**4-Ethylcatechol**	137	122	-80	-22	-5
**2,5-Dihydroxybenzoic acid**	153	108	-50	-18	-15
**4-Methylcatechol**	123	108	-75	-22	-7
**3,4-Dihydroxybenzoic acid**	153	109	-70	-20	-3
**3-Hydroxytyrosol**	153	123	-50	-18	-5
**Kaempferol**	285	151	-85	-26	-7
**Apigenin**	269	117	-95	-48	-7
**Oleuropein**	539	59	-60	-78	-11
**4-Vinylguiacol**	149	121	-125	-16	-9
**trans-Cinnamic acid**	147	103	-50	-16	-17
**Polydatin**	389	227	-85	-20	-19
**Pinocembrin**	255	151	-65	-38	-1
**Tricetin**	301	149	-75	-52	-25
**Galangin**	269	41	-80	-100	-3
**Chrysin**	253	63	-90	-46	-3

Table S6. Physicochemical parameters for the analyzed honeys from each country.

		pH	Conductivity (µS cm^{-1})	Brix index (°Bx)	Water content (%)
**Spain**	SP1	4.37	426.0	80.4	18.0
	SP2	3.76	335.0	82.2	16.2
	SP3	3.85	219.0	80.2	18.2
	SP4	3.72	178.3	79.8	18.6
	SP5	3.91	444.0	83.4	15.0
	SP6	4.31	592.0	82.0	16.4
	SP7	3.95	329.0	79.4	19.0
	SP8	3.68	155.2	81.6	16.8
	SP9	3.91	457.0	80.8	17.6
	SP10	3.88	138.1	81.0	17.4
**Mean ± SD**		3.9 ± 0.2	300± 200	81 ± 1	17 ± 1
**Italy**	IT1	3.77	477.0	79.2	19.2
	IT2	3.66	202.0	81.8	16.6
	IT3	3.54	307.0	81.0	17.4
	IT4	3.64	110.5	80.0	18.2
	IT5	3.56	235.0	80.4	18.0
	IT6	3.68	108.8	80.8	17.6
	IT7	3.53	137.4	80.8	17.6
	IT8	3.67	341.0	80.2	18.2
	IT9	3.68	353.0	80.8	17.6
	IT10	3.73	121.0	80.8	17.6
**Mean ± SD**		3.65 ± 0.08	200 ± 100	80.6 ± 0.7	17.8 ± 0.7
**France**	FR1	4.02	127.7	81.2	17.2
	FR2	4.06	125.6	81.2	17.2
	FR3	4.11	413.0	81.8	16.6
	FR4	4.16	407.0	81.6	16.8
	FR5	4.51	711.0	81.2	17.2
	FR6	4.57	702.0	81.2	17.2
**Mean ± SD**		4.2 ± 0.2	400 ± 300	81.4 ± 0.3	17.0 ± 0.3
**Japan**	JP4	3.97	148.7	77.4	21.0
	JP5	3.75	90.5	79.8	18.6
	JP8	3.37	120.2	80.4	18.0
	JP9	3.58	184.2	80.8	17.6
	JP10	3.96	113.0	81.0	17.4
	JP11	3.83	186.3	78.0	20.4
	JP15	3.79	145.1	78.8	19.6
	JP16	3.54	152.5	79.4	19.0
	JP17	3.61	128.6	79.6	18.8
	JP18	3.32	197.6	80.4	18.0

**Mean ± SD**		3.7 ± 0.2	150 ± 40	80 ± 1	19 ± 1
**Portugal**	PT1	4.00	548.0	82.2	16.2
	PT2	3.79	141.8	82.8	15.6
	PT3	3.76	272.0	82.6	15.8
	PT4	3.79	154.1	82.2	16.2
	PT5	3.59	199.4	81.2	17.2
**Mean ± SD**		3.8 ± 0.2	300 ± 200	82.2 ± 0.6	16.2 ± 0.6
**Serbia**	SE1	3.70	111.8	81.2	17.2
	SE2	4.05	329.0	80.6	17.8
	SE3	4.18	626.0	82.2	16.2
	SE4	3.70	279.0	82.0	16.4
	SE5	3.72	179.9	82.2	16.2
	SE6	3.70	115.5	81.8	16.6
	SE7	3.70	291.0	82.0	16.4
	SE8	3.76	186.8	80.8	17.6
	SE9	3.68	149.3	80.8	17.6
	SE10	3.88	274.0	81.8	16.6
**Mean ± SD**		3.8 ± 0.2	300 ± 200	81.5 ± 0.6	16.9 ± 0.6
**Australia**	AU2	3.87	353.0	82.0	16.4
	AU3	3.76	368.0	80.8	17.6
	AU4	3.72	238.0	80.8	17.6
	AU5	3.96	283.0	81.2	17.2
	AU6	3.93	165.1	83.4	15.0
	AU7	4.08	363.0	83.0	15.4
	AU8	4.13	334.0	81.4	17.0
	AU9	4.10	180.2	82.4	16.0
	AU10	4.10	230.0	82.6	15.8
	AU11	3.74	179.7	82.2	16.2
	AU12	4.27	467.0	82.0	16.4
	AU13	3.80	347.0	81.6	16.8
**Mean ± SD**		4.0 ± 0.2	290 ± 90	82.0 ± 0.8	16.5 ± 0.8
**China**	CH1	3.73	116.5	80.4	18.0
	CH2	3.76	103.6	81.4	17.0
	CH3	3.83	91.4	79.8	18.6
	CH4	3.77	97.2	81.4	17.0
	CH5	3.76	96.0	81.0	17.4
**Mean ± SD**		3.77 ± 0.04	100 ± 10	80.8 ± 0.7	17.6 ± 0.7

Table S7. TPC (total phenolic content), TFC (total flavonoid content), antioxidant capacity (FRAP), and sugar content of the honeys under study.

		TPC (mg eq. gallic acid / g honey)	TFC (mg eq. quercetin / g honey)	FRAP (mg eq. Trolox / g honey)	DNS (mg eq. glucose/g honey)
**Spain**	SP1	0.81	4.17	0.57	618
	SP2	0.69	4.05	0.50	574
	SP3	0.55	2.02	0.16	612
	SP4	0.47	1.62	0.20	582
	SP5	0.95	4.58	0.55	590
	SP6	1.39	8.60	0.87	498
	SP7	0.75	2.97	0.38	535
	SP8	0.51	2.16	0.23	498
	SP9	1.51	3.59	0.58	516
	SP10	0.67	2.53	0.27	514
**Mean ± SD**		0.8 ± 0.4	3 ± 2	0.4 ± 0.2	550 ± 50
**Italy**	IT1	0.68	3.57	0.37	426
	IT2	0.64	3.00	0.47	532
	IT3	0.64	4.66	0.70	518
	IT4	0.34	0.99	0.14	659
	IT5	0.75	3.30	0.10	548
	IT6	0.39	0.79	0.39	527
	IT7	0.42	1.79	0.12	546
	IT8	1.00	4.53	0.49	486
	IT9	1.08	3.29	0.46	494
	IT10	0.46	2.23	0.47	516
**Mean ± SD**		0.6 ± 0.3	3 ± 1	0.4 ± 0.2	530 ± 60
**France**	FR1	0.57	0.93	0.16	517
	FR2	0.28	0.96	0.13	560
	FR3	0.53	2.38	0.36	483
	FR4	0.59	2.90	0.35	516
	FR5	0.79	1.49	0.66	469
	FR6	0.79	1.74	0.59	483
**Mean ± SD**		0.6 ± 0.2	1.7 ± 0.8	0.4 ± 0.2	500 ± 30
**Japan**	JP4	0.32	0.99	0.27	507
	JP5	0.18	0.19	0.11	512
	JP8	0.17	0.48	0.36	517
	JP9	0.24	0.89	0.17	522
	JP10	0.29	0.55	0.22	478
	JP11	0.32	0.87	0.21	550
	JP15	0.46	1.37	0.38	502
	JP16	0.24	0.69	0.11	477
	JP17	0.34	0.65	0.19	509

	JP18	0.43	0.57	0.37	501
**Mean ± SD**		0.3 ± 0.1	0.7 ± 0.3	0.2 ± 0.1	510 ± 20
**Portugal**	PT1	1.18	3.63	1.02	478
	PT2	0.26	1.49	0.15	550
	PT3	0.60	3.34	0.66	456
	PT4	0.33	1.93	0.17	595
	PT5	0.44	2.22	0.23	597
**Mean ± SD**		0.6 ± 0.4	2.5 ± 0.9	0.4 ± 0.4	540 ± 70
**Serbia**	SE1	0.34	1.76	0.10	575
	SE2	0.36	1.57	0.14	484
	SE3	1.43	8.31	0.49	517
	SE4	0.89	11.61	0.17	552
	SE5	0.51	1.72	0.15	526
	SE6	0.57	0.56	0.17	556
	SE7	0.41	1.87	0.19	514
	SE8	0.45	1.59	0.14	522
	SE9	0.39	1.13	0.12	512
	SE10	0.44	2.20	0.19	530
**Mean ± SD**		0.6 ± 0.3	3 ± 3	0.2 ± 0.1	530 ± 30
**Australia**	AU2	0.38	1.07	0.20	518
	AU3	0.45	1.92	0.28	524
	AU4	0.66	2.20	0.33	519
	AU5	0.78	2.63	0.32	561
	AU6	0.47	1.44	0.21	563
	AU7	0.79	2.46	0.37	555
	AU8	0.60	2.02	0.30	544
	AU9	0.45	2.60	0.21	547
	AU10	0.83	2.84	0.45	547
	AU11	0.77	1.51	0.12	533
	AU12	0.47	2.28	0.33	537
	AU13	0.74	2.38	0.47	517
**Mean ± SD**		0.6 ± 0.2	2.1 ± 0.5	0.3 ± 0.1	540 ± 20
**China**	CH1	0.30	1.28	0.15	535
	CH2	0.27	1.07	0.13	595
	CH3	0.20	0.44	0.07	503
	CH4	0.26	1.19	0.13	563
	CH5	0.22	0.77	0.07	568
**Mean ± SD**		0.25 ± 0.04	1.0 ± 0.3	0.11 ± 0.04	550 ± 40
